# Analysis of *RPGR* gene mutations in 41 Chinese families affected by X-linked inherited retinal dystrophy

**DOI:** 10.3389/fgene.2022.999695

**Published:** 2022-10-06

**Authors:** Xiaozhen Liu, Ruixuan Jia, Xiang Meng, Likun Wang, Liping Yang

**Affiliations:** ^1^ Department of Ophthalmology, Peking University Third Hospital, Beijing, China; ^2^ Beijing Key Laboratory of Restoration of Damaged Ocular Nerve, Peking University Third Hospital, Beijing, China; ^3^ Institute of Systems Biomedicine, Department of Pathology, School of Basic Medical Sciences, Beijing Key Laboratory of Tumor Systems Biology, Peking-Tsinghua Center of Life Sciences, Peking University Health Science Center, Beijing, China

**Keywords:** inherited retinal dystrophies, female carriers, hereditary eye disease enrichment panel, *RPGR* gene, *in vitro* splice assay

## Abstract

**Background:** This study analyzed the phenotypes and genotypes of 41 Chinese families with inherited retinal dystrophy (IRD) and *RPGR* gene mutations.

**Methods:** This retrospective analysis evaluated a cohort of 41 patients who were subjected to a specific Hereditary Eye Disease Enrichment Panel (HEDEP) analysis. All (likely) pathogenic variants were determined by Sanger sequencing, and co-segregation analyses were performed on the available family members. All cases were subjected to Sanger sequencing for *RPGR* open reading frame 15 (ORF15) mutations.

**Results:** A total of 41 probands from different families with a clinical diagnosis of retinitis pigmentosa (RP; 34 cases) and cone-rod dystrophy (CORD; 7 cases) were included in this cohort. According to clinical information, 2, 18, and 21 cases were first assigned as autosomal dominant (AD), sporadic, and X-linked (XL) inheritance, respectively. Several cases of affected females who presented with a male phenotype have been described, posing challenges at diagnosis related to the apparent family history of AD. Mutations were located in *RPGR* exons or introns 1–14 and in ORF15 of 12 of 41 (29.3%) and 29 of 41 (70.7%) subjects, respectively. Thirty-four (likely) pathogenic mutations were identified. Frameshifts were the most frequently observed variants, followed by nonsense, splice, and missense mutations. Herein, a detailed description of four RP patients carrying *RPGR* intronic mutations is reported, and *in vitro* splice assays were performed to confirm the pathogenicity of these intronic mutations.

**Conclusion:** Our findings provide useful insights for the genetic and clinical counseling of patients with XL IRD, which will be useful for ongoing and future gene therapy trials.

## Introduction

X-linked retinitis pigmentosa (XL RP) is the most severe subtype of RP (OMIM 268000) and accounts for 10%–20% of all RP cases (Breuer et al., 2002; Bader et al., 2003). Male patients with XL RP generally develop a more severe phenotype, whereas female carriers of XL RP show a wide spectrum of clinical features that can vary from asymptomatic to severe symptoms similar to those observed in male patients (Wu et al., 2010; Nanda et al., 2018; Talib et al., 2018). At present, mutations in three genes, *RP*2 (OMIM #312600), O*FD1* (OMIM #312610), and *RPGR* (OMIM #312610), have been reported to result in XL RP (Breuer et al., 2002; Bader et al., 2003). Among them, *RPGR* variations remain the most common genetic cause, and are found in approximately 30%–80% of XL RP cases (Vervoort et al., 2000; Pelletier et al., 2007). Previous studies have indicated that *RPGR* variants are also responsible for other XL inherited retinal dystrophies (IRDs), including cone dystrophy 3(COD3, OMIM #602093), cone-rod dystrophy 2 (CORD2, OMIM #120970) (Mears et al., 2000; Ebenezer et al., 2005), and macular degeneration (Ayyagari et al., 2002).

*RPGR* was previously identified in the RP3 region of Xp11.4 (Meindl et al., 1996; Roepman et al., 1996). The two main isoforms are the constitutively expressed *RPGR* ^*1-19*^ isoform, which consists of 19 exons, and the retina-specific *RPGR* ^*ORF15*^ isoform, which shares exons 1 to 14 with *RPGR* ^*1-19*^, and includes a terminal exon (open reading frame 15, ORF15). ORF15 consists of exon 15 and extends into intron 15, encoding a highly repetitive and purine-rich 567-aa protein. Approximately 60% of disease-causing mutations in *RPGR* are found in ORF15 (Vervoort et al., 2000; Breuer et al., 2002).

This study reports the clinical and genetic findings in a Chinese cohort of 40 male probands and a female proband with RP (34 cases) or CORD (7 cases) caused by variations in *RPGR.* Among them, we report two families with a provisional diagnosis of autosomal dominant RP (ADRP) associated with affected female carriers and characterized by a phenotypic manifestation in these patients. We describe four patients with RP who carried *RPGR* intronic mutations in detail and confirmed the pathogenicity of these intronic mutations using an *in vitro* splice assay.

## Materials and methods

### Patients

This study recruited 40 male Chinese probands and a female proband of Han ethnicity affected with IRD, including RP and CORD, whose genetic causes were *RPGR* mutations, from the Department of Ophthalmology, Peking University Third Hospital. This study conforms to the tenets of the Declaration of Helsinki. All experiments involving DNA from a patient and their relatives were approved by the Peking University Third Hospital Medical Ethics Committee (No. 2012093). Written informed consent was obtained from all participants or guardians on behalf of minors. The ethics committee approved the consent procedure.

Detailed medical and family histories were obtained from probands and/or their family members. All patients underwent standard ophthalmic examinations, including best-corrected visual acuity (BCVA), slit-lamp biomicroscopy, intraocular pressure measurement, dilated indirect ophthalmoscopy and fundus photography, electroretinography (ERG), and visual field tests, if possible. The patients underwent a systematic physical examination before genetic testing. Inheritance patterns were classified based on criteria described by Stone et al. with minimal modification (Stone et al., 2017): (i) autosomal dominant (AD; a minimum of three generations with at least one instance of male-to-male transmission); (ii) autosomal recessive (AR; several affected patients in a single sibship with healthy parents); (iii) X-linked (affected males in multiple sibships connected to each other through unaffected or mildly affected females and no male-to-male transmission); and (iv) uncertain inheritance (sporadic patients and other multiplex kindreds).

### Molecular genetics analysis

Blood samples were obtained from all probands and their family members. Genomic DNA (gDNA) was extracted using standard protocols ((D2492, Omega Bio-Tek). The patients were subjected to a specific hereditary eye disease enrichment panel (HEDEP) for targeted exon enrichment analysis, which captured 483 IRD genes (Liu et al., 2021). Next-generation sequencing (NGS) was performed using an Illumina HiSeq X platform (Illumina, San Diego, CA, USA). HEDEP sequencing data were analyzed as previously described (Zhang et al., 2016; Liu et al., 2021).

### Sanger sequencing and mutation screening of *RPGR* ORF15

All variants considered (likely) pathogenic by NGS in this study were validated further by PCR-based Sanger sequencing. The primers used in this study are listed in Supplementary Table S1. Touchdown PCR amplification consisted of a denaturizing step at 95°C for 5 min, followed by 35 cycles of amplification (at 95°C for 30 s, at 64∼57°C for 30 s starting from 64°C, decreasing by 0.5°C every cycle for 14 cycles, which was maintained at 57°C for 21 cycles, followed by 72°C for 40 s), with a final extension at 72°C for 5 min. Purified PCR products were sequenced using the ABI BigDye Terminator cycle sequencing kit on an ABI 3130XL genetic analyzer (ABI Applied Biosystem, Foster City, CA, USA). Sanger sequencing was also used to determine whether the variant co-segregated with the disease phenotype in available family members.

*RPGR* ORF15 (NG 009553.1; NM001034853) was not effectively covered by NGS and was therefore amplified and sequenced using primers located outside the repetitive stretch as described previously (Bader et al., 2003). The forward primer was 5′-CAG​AGA​TCC​TAT​CAG​ATG​ACC-3′, and the reverse primer was 5′-TGT​CTG​ACT​GGC​CAT​AAT​CG-3′, with a PCR product of 1630 bp. PCR products were sequenced using three reverse primers (5′-GTT​TGC​CAT​ATT​TCA​CAG​ATC​C-3′, 5′-TCC​TTC​CTC​CTC​TTC​CCC​CTC​CCA-3′, and 5′-CCT​TCC​TCC​TCT​TCC​CCC​TCA-3′). Sequencing results were analyzed by Sequencher (Gene Codes Corporation, Ann Arbor, MI, USA).

### High throughput data analysis and variant classification

HEDEP sequencing data were analyzed as described previously (Liu et al., 2021). Sequence changes were classified according to the American College of Medical Genetics and Genomics and the Association for Molecular Pathology variant interpretation guidelines. In this study, only variants identified as pathogenic or likely pathogenic were reported.

### Splice prediction analysis

The pathogenicity of *RPGR* intronic mutations was assessed using a population-based genome dataset (the Single Nucleotide Polymorphism Database). The potential effect of the variants on RNA splicing was assessed using the Human Splice Finder (HSF) tool (Desmet et al., 2009).

### *In vitro* splice assay

For use in the RNA splicing assay, a shortened *RPGR* intron DNA sequence around the splice acceptor site (intron A, 200 bp) was generated and combined with another shortened intron DNA sequence around the splice donor site (intron B, 200 bp). Introns A and B were combined to generate a shortened *RGPR* intron. The plasmid pmCherryN1 was linearized using the *Sbf*I restriction enzyme (R3733S, New England Biolabs), followed by purification with a gel extraction kit (D2500, Omega). The shortened *RGPR* intron was cloned into the *Sbf*I site using the pEASY-Basic Seamless Cloning and Assembly Kit (CU201, TransGen Biotech) and then transformed into Top10-competent bacteria. Clones were amplified in liquid Luria-Bertani (LB) broth, followed by the extraction of plasmid DNA (D6943; OMEGA). Site-directed mutagenesis was performed on the plasmid using the StarMut Site-directed Mutagenesis Kit (T111, Genstar, Beijing, China). The resulting DNA was sequenced to confirm nucleotide changes. HEK293T cells were cultured in regular Dulbecco’s modified Eagle’s medium (DMEM; C11995500BT, Gibco) and seeded on coated glass slides in 6-well plates. The cells were transfected with recombinant vectors using PEI (B600070, ProteinTech Group, Chicago, IL, USA), according to the manufacturer’s protocol. After 24 or 72 h of transfection, mCherry expression was observed using a fluorescence microscope (Nikon, Japan).

### Statistical analysis

The results were evaluated using a two-tailed unpaired Student’s *t*-test and are presented as the mean ±SEM. GraphPad Prism 9 (GraphPad Software, La Jolla, CA, USA) was used for the statistical analyses. Statistical significance was set at *p* < 0.05.

## Results

### Clinical phenotypes of the Chinese *RPGR*-IRD cohort

In this cohort, 40 male Chinese probands and one female proband from 41 families with a clinical diagnosis of RP (34 cases) or CORD (7 cases) were found to harbor a disease-causing mutation in the *RPGR* gene. Table 1 provides an overview of the cohort’s primary clinical information. According to their clinical information, the inheritance pattern of 2 cases was assigned as AD without male-to-male transmission, with 18 cases assigned as sporadic, and 21 cases assigned as XL. Apart from the proband of family P25 (proband P25), whose onset age was 20 years, the average age at the onset of the first symptom was 4.85 years (range = 1∼11 years). Apart from proband P33, all probands complained that night blindness was present at disease onset, and three patients suffering from CORD, simultaneously complained of poor visual acuity. Twenty-seven patients had progressive visual acuity. Eighteen patients at study entry experienced a sudden decline in central vision in their 20 and 30s, with a BCVA for light perception (LP) of 0.25. Three patients suffered from unilateral or bilateral blindness; among them, the left eye of proband P11 was blind owing to untreated retinal detachment when he was 14, the right eye of proband P31 was blind when he was 50, and proband P31 suffered from bilateral blindness when he was 60. Fundus examination and imaging results of all probands are listed in Table 1. Four probands (P09, P11, P27, and P31) complained of high myopia; the mothers of probands P09 and P11, as carriers, also suffered from high myopia and/or poor visual acuity. The mother of proband P18, who was diagnosed as an *RPGR* carrier, also complained of high myopia.

**TABLE 1:** Clinical information of 41 Chinese X-linked inherited retinal dystrophy families caused by *RPGR* mutations.

Family ID	Gender	Clinical diagnosis	Inheritance from phenotype	Family history	Age (years)		BCVA		First symptoms	Cataract(age)	Myopia			Fundus appearance					ERG
					At entry	Onset	L.E. (age)	R.E. (age)			L.E.		R.E.	RPE degeneration	OD	AA	PD	MD	
P01	M	RP	Sporadic	NO	45	7	0.01	0.01	Night blindness	Yes (38)		No		Widespread	Waxy	Yes	Mid-periphery	Severe	Diminished
P02	M	RP	Sporadic	NO	12	3	0.6	0.6	Night blindness	No		No		No	Rudy	No	Mid-periphery	Severe	Significantly reduced
P03	M	RP	Sporadic	NO	13	4	0.5	0.5	Night blindness	No		No		Mild	Rudy	No	Mid-periphery	Severe	Significantly reduced
P04	M	RP	XL	Yes	32	6	0.3	0.4	Night blindness	No		No		Widespread	Waxy	Yes	Mid-periphery	Severe	Diminished
P05	M	RP	XL	Yes	28	5	0.4	0.4	Night blindness	No		No		Mild	Waxy	Yes	Mid-periphery	Severe	Diminished
P06	M	RP	XL	Yes	40	8	0.1	0.1	Night blindness	Yes (35)		No		Widespread	Rudy	Yes	Mid-periphery	Severe	Diminished
P07	M	RP	XL	Yes	30	5	0.2	0.2	Night blindness	No		No		Widespread	Rudy	Yes	Mid-periphery	Severe	Diminished
P08	M	RP	Sporadic	NO	9	4	0.7	0.7	Night blindness	No		No		No	Rudy	No	Mid-periphery	Mild	Mildly reduced
P09	M	RP	Sporadic	Yes	14	3	0.4	0.4	Night blindness	No	(-10D)		(-10D)	Mild	Rudy	No	Mid-periphery	Severe	Significantly reduced
P10	M	RP	XL	Yes	10	2	0.6	0.5	Night blindness	No		No		No	Rudy	No	Mid-periphery	Mild	Mildly reduced
P11	M	RP	Sporadic	Yes	17	3	blind (15)	0.4	Night blindness	No		-	(-17D)	Widespread	Rudy	Yes	Mid-periphery	Severe	Significantly reduced
P12	M	CORD	XL	Yes	10	4	0.7	0.7	Night blindness	No		No		No	Rudy	No	Periphery	Mild	Mildly reduced
P13	M	RP	Sporadic	NO	9	3	0.6	0.6	Night blindness	No		No		No	Rudy	No	Mid-periphery	Mild	Mildly reduced
P14	M	RP	Sporadic	NO	31	5	0.3	0.3	Night blindness	No		No		Widespread	Waxy	Yes	Mid-periphery	Severe	Diminished
P15	M	RP	XL	Yes	30	5	0.1	0.1	Night blindness	No		No		No	Waxy	Yes	Mid-periphery	Severe	Diminished
P16	M	RP	XL	Yes	7	3	0.6	0.6	Night blindness	No		No		No	Rudy	No	Mid-periphery	Mild	Mildly reduced
P17	M	RP	XL	Yes	35	7	0.1	0.1	Night blindness	No		No		Widespread	Waxy	Yes	Mid-periphery	Severe	Diminished
P18	M	RP	Sporadic	Yes	11	3	0.1	0.1	Night blindness	No		No		No	Rudy	No	Periphery	Severe	Significantly reduced
P19	M	RP	AD	Yes	27	5	0.3	0.3	Night blindness	No		No		No	Rudy	Yes	Whole	Severe	Diminished
P20	M	RP	AD	Yes	36	5	0.25	0.25	Night blindness	No		No		Widespread	Waxy	Yes	Mid-periphery	Severe	Diminished
P21	M	RP	Sporadic	NO	39	6	0.1	0.1	Night blindness	No		No		Widespread	Waxy	Yes	Mid-periphery	Severe	Diminished
P22	M	RP	XL	Yes	45	6	0.01	0.01	Night blindness	Yes		No		Widespread	Waxy	Yes	Mid-periphery	Severe	Diminished
P23	M	RP	XL	Yes	43	7	0.01	0.01	Night blindness	Yes (40)		No		Widespread	Waxy	Yes	Whole	Severe	Diminished
P24	M	RP	XL	Yes	38	5	0.1	0.1	Night blindness	Yes (37)		No		Widespread	Waxy	Yes	Mid-periphery	Severe	Diminished
P25	M	RP	XL	Yes	40	20	0.3	0.1	Night blindness	Yes (35)		No		Mild	Waxy	Yes	Whole	Severe	Diminished
P26	M	RP	XL	Yes	39	6	0.1	LP	Night blindness	Yes (37)		No		Widespread	Waxy	Yes	Whole	Severe	Diminished
P27	F	RP	XL	Yes	68	4	HM	LP	Night blindness	Yes (54)	(-7D)		(-7D)	Widespread	Waxy	Yes	Whole	Severe	Diminished
P28	M	RP	XL	Yes	36	3	0.4	0.4	Night blindness	No		No		Widespread	Waxy	Yes	Mid-periphery	Severe	Diminished
P29	M	RP	XL	Yes	42	5	0.1	0.1	Night blindness	No		No		Widespread	Waxy	Yes	Whole	Severe	Diminished
P30	M	RP	Sporadic	NO	44	3	0.1	0.1	Night blindness	No		No		Widespread	Waxy	Yes	Mid-periphery	Severe	Diminished
P31	M	RP	Sporadic	NO	50	4	0.3	blind(50)	Night blindness	Yes (46)	(-8D)		(-8D)	Widespread	Waxy	Yes	Whole	Severe	Diminished
P32	M	CORD	Sporadic	NO	16	2	0.4	0.4	Night blindness; Poor visual acuity	No		No		Mild	Waxy	Yes	Mid-periphery	Severe	Significantly reduced
P33	M	CORD	XL	Yes	64	3	blind(60)	blind(60)	Poor visual acuity	Yes (50)		No		Widespread	Waxy	Yes	Whole	Severe	Diminished
P34	M	CORD	XL	Yes	45	11	0.02	0.02	Night blindness; Poor visual acuity	Yes (40)		No		Widespread	Waxy	Yes	Whole	Severe	Diminished
P35	M	CORD	XL	Yes	34	5	0.3	0.3	Night blindness; Poor visual acuity	No		No		Widespread	Waxy	Yes	Mid-periphery	Severe	Diminished
P36	M	CORD	Sporadic	NO	50	10	0.1	0.1	Night blindness	Yes (40)		No		Widespread	Waxy	Yes	Whole	Severe	Diminished
P37	M	RP	Sporadic	NO	46	1	0.3	0.3	Night blindness	Yes (42)		No		Widespread	Waxy	Yes	Mid-periphery	Severe	diminished
P38	M	RP	XL	Yes	6	3	0.6	0.6	Night blindness	No		No		No	Rudy	No	Periphery	Mild	Mildly reduced
P39	M	CORD	Sporadic	NO	46	3	0.01	0.01	Night blindness	Yes (38)		No		Widespread	Waxy	Yes	Mid-periphery	Severe	Diminished
P40	M	RP	Sporadic	NO	12	10	0.2	0.2	Night blindness	No		No		No	Rudy	No	Periphery	Severe	Significantly reduced
P41	M	RP	Sporadic	NO	35	7	0.3	0.3	Night blindness	No		No		Widespread	Waxy	Yes	Whole	Severe	Diminished

BCVA, best corrected visual acuity; HM, hand movement; LP, light perception; L.E., left eyes; F, female; M, male; MD, macular degeneration; R.E., right eyes; XL, X-linked; RP, retinitis pigmentosa; CORD, cone-rod dystrophy; OD, optic disc; AA, artery attenuation; PD, pigment deposits. Age at entry corresponded to the age of the first visit to the center; onset age was defined as the self-reported age of the patient’s first symptoms.

### Molecular diagnosis of the Chinese *RPGR*-IRD cohort

We identified *RPGR* (likely) causative variants in 41 Chinese probands affected by RP or CORD (Table 2). All samples were subjected to HEDEP analysis, and (likely) pathogenic variants in the *RPGR* exons or introns 1–14 were identified in 12 patients. All patients in this cohort were subjected to direct PCR-based Sanger sequencing of the entire *RPGR* ORF15. We identified (likely) pathogenic variants of *RPGR* ORF15 in 29 unsolved cases. All identified *RPGR* variants were segregated with IRD phenotypes among the available family members. Proband P27 had *RPGR* variants that might have been inherited from her affected father; however, her father was not subjected to HEDEP or other genetic testing methods. The other 40 male probands had *RPGR* variants inherited from their mothers. Thirty-four mutations were identified in this cohort (Table 2), of which one was novel and 21 were first reported in our previous study (Liu et al., 2021). In this study, 29 patients carried variants in *RPGR* ORF15, 4 had intronic variants, and 8 harbored variants in exons 1–14. In terms of the type of mutation, frameshifts were the most frequently observed variants in this cohort (*n* = 15), followed by nonsense mutations (*n* = 13), splice variants (*n* = 4), and missense mutations (*n* = 2).

**TABLE 2:** Genetic Diagnosis of 41 Chinese XLRP families caused by *RPGR* mutations.

Family ID	Exon	HGVS g. Name (nucleotide changes)	HGVS c. Name (nucleotide changes)	HGVS p. Name (amino acid change)	ClinVar IDs	Classification	gnomAD_Allele frequency	Reported/novel
P01	IVS4	NC_000023.10:g.38180277T>C	NM_000328.3:c.310 + 3A>G	p.?	98780	Pathogenic	NA	Yes
P02	IVS1	NC_000023.10:g.38182779T>A	NM_000328.3:c.29-2A>T	p.?	NA	Likely pathogenic	NA	Yes
P03	IVS6	NC_000023.10:g.38176567A>T	NM_000328.3:c.619 + 2T>A	p.?	975129	Likely pathogenic	NA	Yes
P04	IVS14	NC_000023.10:g.38146501G>C	NM_000328.3:c.1754-3C>G	p.?	1065689	likely pathogenic	NA	Yes
P05	ORF15	NC_000023.10:g.38146066_38146079del	NM_001034853.2:c.2173_2186del	p.Gln725Glyfs*40	NA	Pathogenic	NA	Yes
P06	E13	NC_000023.10:g.38150243G>C	NM_000328.3:c.1541C>G	p.Ser514*	1213922	Pathogenic	NA	Yes
P07	ORF15	NC_000023.10:g.38146087_38146091del	NM_001034853.2:c.2164_2168del	p.Glu722Lysfs*46	NA	Pathogenic	NA	Yes
P08	ORF15	NC_000023.10:g.38145557C>A	NM_001034853.2:c.2695G>T	p.Glu899*	866316	Pathogenic	NA	Yes
P09	E11	NC_000023.10:g.38156552G>A	NM_000328.3:c.1399C>T	p.Gln467*	NA	Pathogenic	NA	Yes
P10	ORF15	NC_000023.10:g.38146018_38146019del	NM_001034853.2:c.2236_2237del	p.Glu746Argfs*23	438142	Pathogenic	NA	Yes
P11	ORF15	NC_000023.10:g.38145848_38145849del	NM_001034853.2:c.2405_2406del	p.Glu802Glyfs*32	91389	Pathogenic	NA	Yes
P12	ORF15	NC_000023.10:g.38145848_38145849del	NM_001034853.2:c.2405_2406del	p.Glu802Glyfs*32	91389	Pathogenic	NA	Yes
P13	E14	NC_000023.10:g.38147175_38147176del	NM_000328.3:c.1693_1694del	p.Gln565Argfs*17	NA	Pathogenic	NA	Yes
P14	ORF15	NC_000023.10:g.38145761C>A	NM_001034853.2:c.2491G>T	p.Glu831*	1297121	Pathogenic	NA	Yes
P15	ORF15	NC_000023.10:g.38145977C>A	NM_001034853.2:c.2275G>T	p.Gly759*	NA	Pathogenic	NA	Yes
P16	ORF15	NC_000023.10:g.38146018_38146019del	NM_001034853.2:c.2236_2237del	p.Glu746Argfs*23	438142	Pathogenic	NA	Yes
P17	E5	NC_000023.10:g.38178216C>G	NM_000328.3:c.335G>C	p.Gly112Ala	NA	Pathogenic	NA	Yes
P18	ORF15	NC_000023.10:g.38145848_38145849del	NM_001034853.2:c.2405_2406del	p.Glu802Glyfs*32	91389	Pathogenic	NA	Yes
P19	ORF15	NC_000023.10:g.38145251C>A	NM_001034853.2:c.3001G>T	p.Glu1001*	NA	Pathogenic	NA	Yes
P20	ORF15	NC_000023.10:g.38145523_38145524del	NM_001034853.2:c.2730_2731del	p.Glu911Glyfs*167	NA	Pathogenic	NA	Yes
P21	ORF15	NC_000023.10:g.38145848_38145849del	NM_001034853.2:c.2405_2406del	p.Glu802Glyfs*32	91389	Pathogenic	NA	Yes
P22	E8	NC_000023.10:g.38163971G>C	NM_000328.3:c.851C>G	p.Ser284*	NA	Pathogenic	NA	Yes
P23	ORF15	NC_000023.10:g.38145992C>A	NM_001034853.2:c.2260G>T	p.Glu754*	866844	Likely pathogenic	NA	Novel
P24	ORF15	NC_000023.10:g.38146018_38146019del	NM_001034853.2:c.2236_2237del	p.Glu746Argfs*23	438142	Pathogenic	NA	Yes
P25	ORF15	NC_000023.10:g.38146018_38146019del	NM_001034853.2:c.2236_2237del	p.Glu746Argfs*23	438142	Pathogenic	NA	Yes
P26	ORF15	NC_000023.10:g.38145848_38145849del	NM_001034853.2:c.2405_2406del	p.Glu802Glyfs*32	91389	Pathogenic	NA	Yes
P27	E6	NC_000023.10:g.38176581C>G	NM_000328.3:c.607G>C	p.Ala203Pro	NA	Pathogenic	NA	Yes
P28	ORF15	NC_000023.10:g.38145827_38145828del	NM_001034853.2:c.2426_2427del	p.Glu809Glyfs*25	183262	Pathogenic	NA	Yes
P29	ORF15	NC_000023.10:g.38145848_38145849del	NM_001034853.2:c.2405_2406del	p.Glu802Glyfs*32	91389	Pathogenic	NA	Yes
P30	E11	NC_000023.10:g.38156606G>A	NM_000328.3:c.1345C>T	p.Arg449*	866558	Pathogenic	NA	Yes
P31	E5	NC_000023.10:g.38178178C>A	NM_000328.3:c.373G>T	p.Glu125*	NA	Pathogenic	NA	Yes
P32	ORF15	NC_000023.10:g.38145807_38145810del	NM_001034853.2:c.2442_2445del	p.Gly817Lysfs*2	620582	Pathogenic	NA	Yes
P33	ORF15	NC_000023.10:g.38145746C>A	NM_001034853.2:c.2506G>T	p.Glu836*	NA	Pathogenic	NA	Yes
P34	ORF15	NC_000023.10:g.38145357dup	NM_001034853.2:c.2899dup	p.Glu967Glyfs*112	624397	Pathogenic	NA	Yes
P35	ORF15	NC_000023.10:g.38146018_38146019del	NM_001034853.2:c.2236_2237del	p.Glu746Argfs*23	438142	Pathogenic	NA	Yes
P36	ORF15	NC_000023.10:g.38146110_38146111insAGCTC	NM_001034853.2:c.2144_2145insCTGAG	p.Arg715Serfs*2	NA	Pathogenic	NA	Yes
P37	ORF15	NC_000023.10:g.38145551del	NM_001034853.2:c.2701del	p.Glu901Lysfs*188	NA	Pathogenic	NA	Yes
P38	ORF15	NC_000023.10:g.38145316_38145320del	NM_001034853.2:c.2933_2937del	p.Glu978Glyfs*99	NA	Pathogenic	NA	Yes
P39	ORF15	NC_000023.10:g.38145452C>A	NM_001034853.2:c.2800G>T	p.Glu934*	NA	Pathogenic	NA	Yes
P40	ORF15	NC_000023.10:g.38145750del	NM_001034853.2:c.2506delG	p.Glu836Lysfs*253	NA	Pathogenic	NA	Yes
P41	ORF15	NC_000023.10:g.38146034C>A	NM_001034853.2:c.2218G>T	p.Glu740*	1275779	Pathogenic	NA	Yes

NA, not available.

### Revision of inheritance pattern in two RP families

**FIGURE 1:**
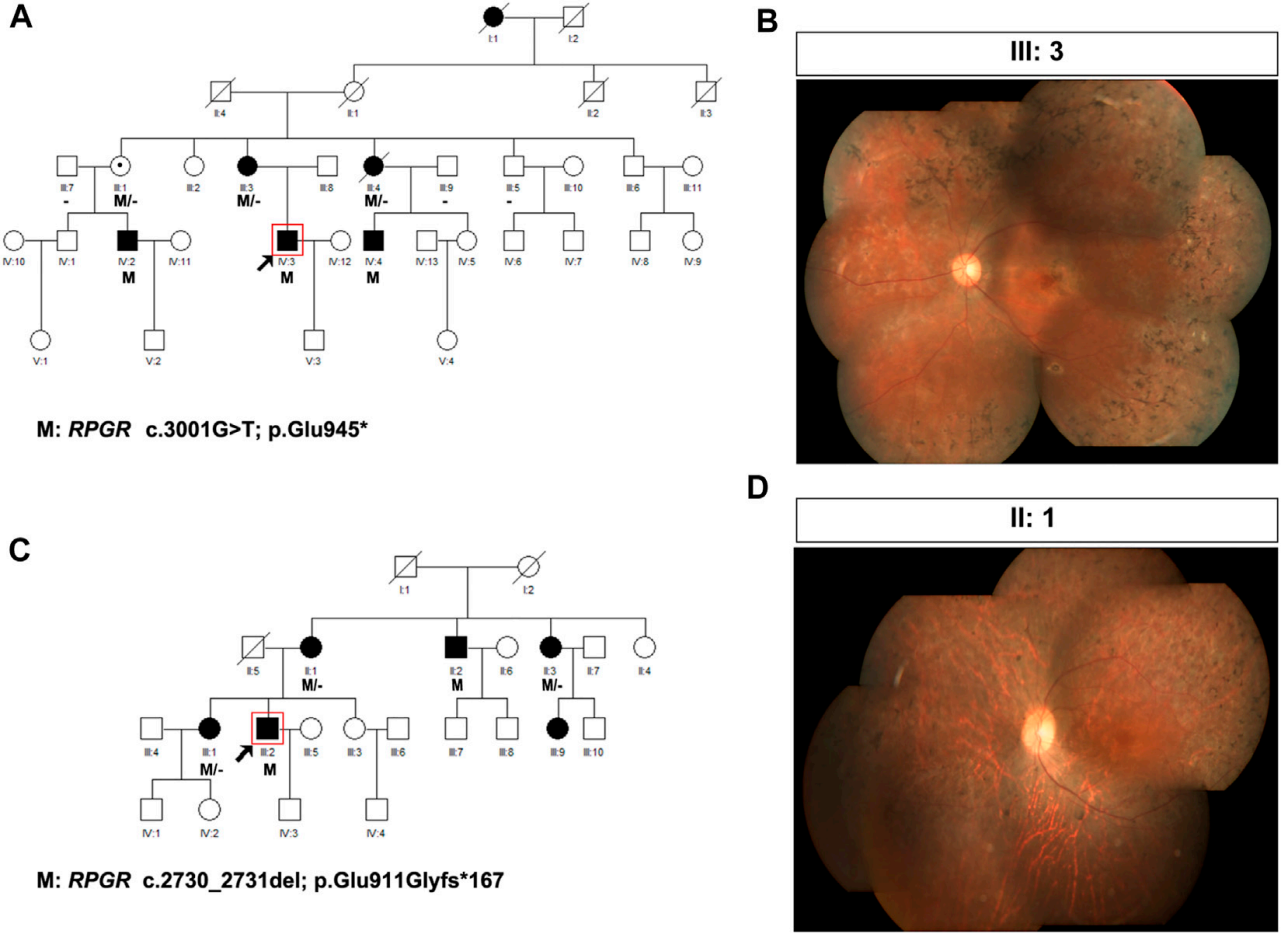
Pedigree and images of families P19 and P20. **(A)** Pedigree of family P19; **(B)** fundus photograph of the left eye of individual III:3 from family P19; **(C)** pedigree of family P20; and **(D)** fundus photograph of the right eye of individual II:1 from family P20. Black squares (males) and circles (females) represent individuals affected with RP. Dotted circles (females) represent individuals who are *RPGR* mutation carriers but who are not affected by RP. Unaffected individuals are not shaded. Black lines indicate deceased individuals The arrow marks the proband.

The presence of affected carriers in XL RP families can misleadingly suggest AD inheritance (De Silva et al., 2021). As illustrated in Figure 1, the pedigrees of patients P19 and P20 showed that males and females were affected, with individuals affected in each generation, suggesting AD inheritance. However, there was no male-to-male transmission; therefore, XL inheritance was not excluded. Genetic testing confirmed that *RPGR* ORF15 variants resulted in RP phenotypes in these two families; therefore, their inheritance pattern was revised to XL.

### Several *RPGR*-related RP carriers with a severe male pattern

In family P19, the proband was a 27-year-old man with night blindness since childhood, progressive decline in visual acuity, tunnel vision, and visual field defects. Sequencing analysis identified c.3001G>T (p. Glu945*), an OFR15 mutation, which was the same pathogenic mutation found in his mother (individual III:3), two aunts (individuals III:1 and III:4), and two affected male cousins (Table 2; Figure 1A). In this family, III:3, an *RPGR* variant carrier, complained of night blindness since childhood, and her BCVA was 0.05 bilaterally. Fundus examination revealed classic symptoms of RP, consisting of retinal arteriolar attenuation, waxy pallor of the optic discs, and scattered bone spicule pigmentation around the mid-peripheral retina or the whole retina (Figure 1B). Individual III:4, a female carrier, also suffered from RP, whereas individual III:1 was unaffected. In family P20, the proband, an RP patient, had an *RPGR* frameshift mutation, c.2730_2731del (p. Glu911Glyfs*167), inherited from his mother (individual II:1). The same pathogenic mutation was also identified in his uncle (individual II:2), aunt (individual II:3), and older sister (individual III:1, Figure 1C). In this family, individual II:1, as an *RPGR* variant carrier, complained of night blindness since childhood and suffered from a sudden decline in vision in her 40s. Fundus examination revealed classic symptoms of RP (Figure 1D). Individuals II:3 and III:1 were female carriers both affected by RP. Another individual, III:9, a 24-year-old woman, also complained of night blindness, although she had not yet undergone a genetic screening test. Clinical diagnosis and genetic testing confirmed that individual II:2 was affected by *RPGR*-related XL RP.

**FIGURE 2:**
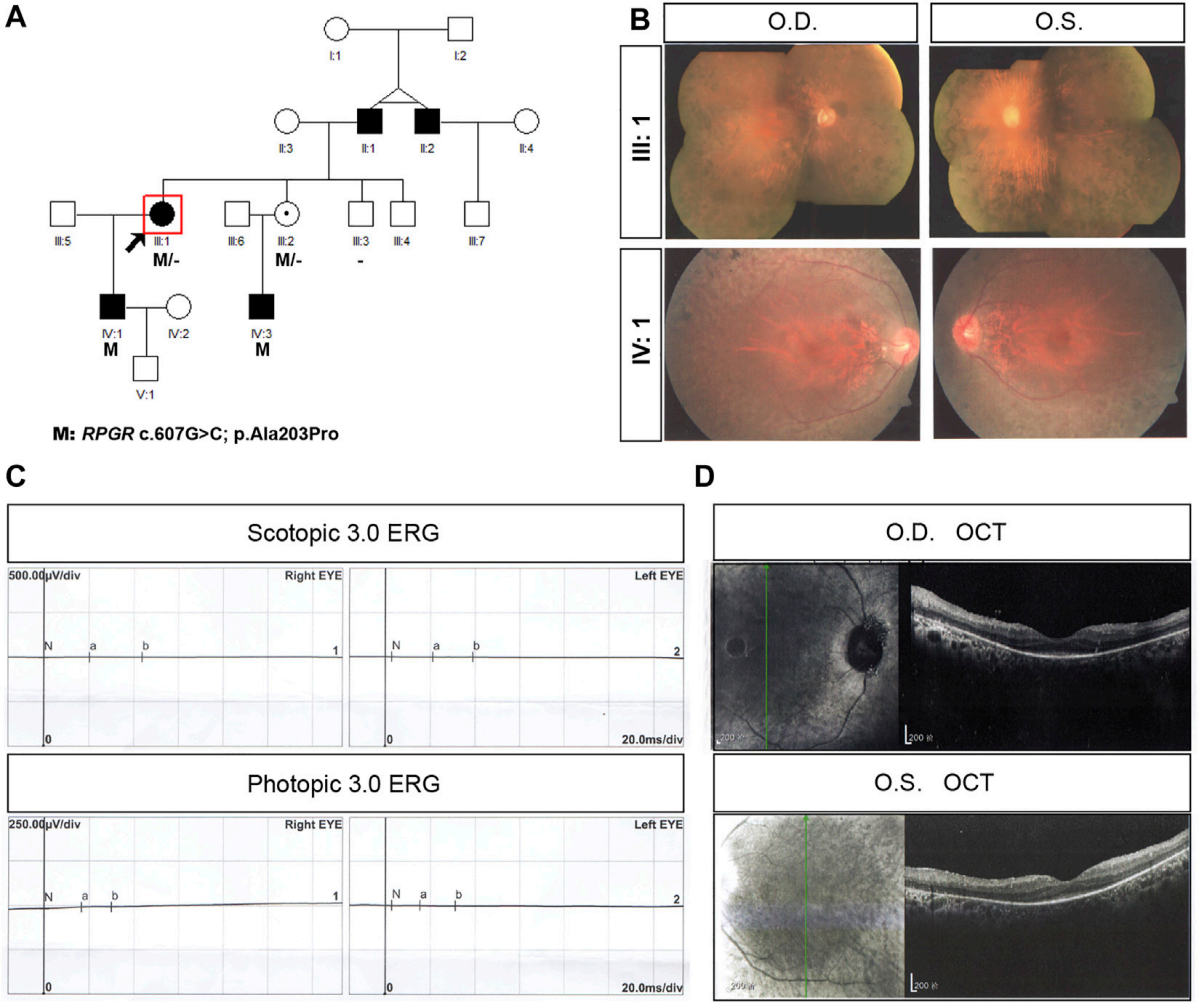
Pedigree and images of family P27. **(A)** Pedigree of family P27; **(B)** fundus photograph of both eyes of the proband (individual III:1) and IV:1 from the family; **(C)** scotopic and photopic electroretinogram results of the proband; and **(D)** F optical coherence tomography image of the both eyes from the proband. Black squares (males) and circles (females) represent individuals affected with RP. Dotted circles (females) represent individuals who are *RPGR* mutation carriers but who are not affected by RP. Unaffected individuals are not shaded. Black lines indicate deceased individuals. The arrow marks the proband.

In family P27, the proband was a 68-year-old woman with end-stage disease. Her vision was hand movement (HM) in the left eye and LP in the right eye (Table 1). This proband began suffering from night blindness at the age of 4 years, and complained of high myopia (-7D) bilaterally. She had undergone cataract surgery bilaterally at the age of 54 years. She had a 40-year-old son (individual IV:1) with the same mutation, c.607G>C (p. Ala203Pro) in exon 6 (Figure 2A). Proband P27 had an XL RP male phenotype fundus appearance similar to that of the affected son (Figure 2B). The proband’s ERG responses were completely extinguished (Figure 2C) and SD-OCT imaging demonstrated thinning of the outer retinal layer (Figure 2D). In this family, several individuals, including her father (individual II:1), the father’s younger twin brother (individual II:2), her son (individual IV:1), and her nephew (individual IV:3) were diagnosed with RP. Sequencing analysis indicated that IV:3 carried the *RPGR* hemizygous variant, c.607G>C (p. Ala203Pro), whereas his mother, as well as the proband’s younger sister (individual III:3), was unaffected, although the younger sister had the same heterozygous variant as the proband.

### Four patients with *RPGR* intronic mutations

**FIGURE 3:**
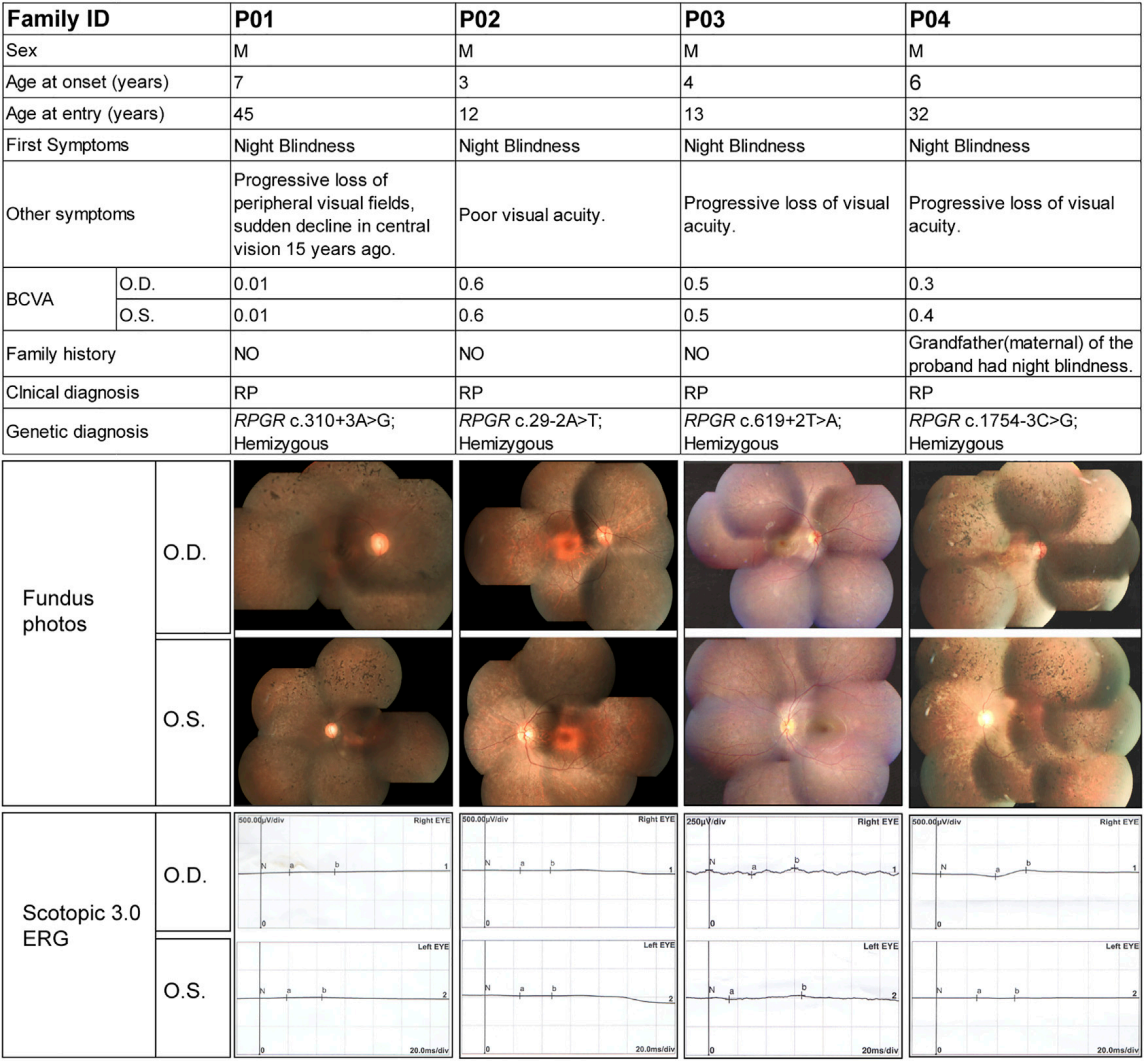
Summary of the clinical and genetic findings of four patients with RP and *RPGR* intronic variants. The composite image shows the most important available clinical findings. Each column represents a single patient with the following information in each row: family ID; sex; age of onset (as reported subjectively by the patient) and at entry; first symptom (first manifestation of the disease as reported by the patient); other symptoms; best corrected visual acuity (BCVA); family history; clinical diagnosis; genotype; fundus images; and scotopic and photopic electroretinogram results. O.D., right eye; O.S., left eye; F, female; M, male.

**FIGURE 4:**
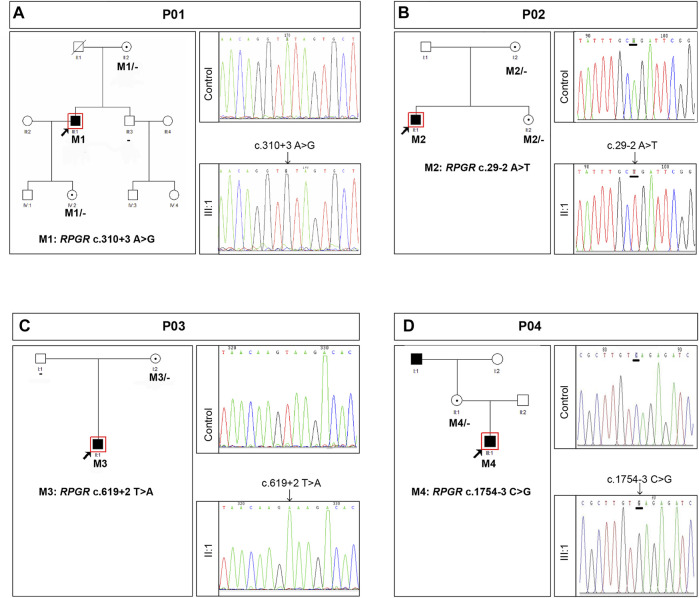
Summary of the genetic findings of four patients with RP and *RPGR* intronic variants. Pedigree and Sanger sequencing results of families P01 **(A)**, P02 **(B)**, P03 **(C)**, and P04 **(D)**. Black squares (males) and circles (females) represent individuals affected with RP. Dotted circles (females) represent individuals who are *RPGR* mutation carriers but who are not affected by RP. Unaffected individuals are not shaded. Black lines indicate deceased individuals. The arrow marks the proband.

Figure 3 shows the clinical information of the four RP patients and their genetic diagnoses. These four probands were male, and their ages ranged from 12 to 45 years old. All probands complained of night blindness since childhood and all had poor visual acuity, with BCVA ranging from 0.01 to 0.6. Probands P01, P03, and P04 suffered from a progressive loss of visual acuity, whereas proband P01 also suffered from a sudden decline in central vision 15 years prior. Probands, except for P04, had no family history of a similar disorder, and the grandfather (maternal) of proband P04 was also affected by night blindness. Fundus examination and imaging revealed the classic symptoms of RP, and full-field ERG showed markedly reduced rod responses in all patients. The observed phenotypes were consistent with a diagnosis of RP in all patients. Genetic analysis showed that all probands had intronic variants in the *RPGR* gene, which were inherited from their mother (Figure 4), although she did not manifest any symptoms despite being an *RPGR* carrier.

**FIGURE 5:**
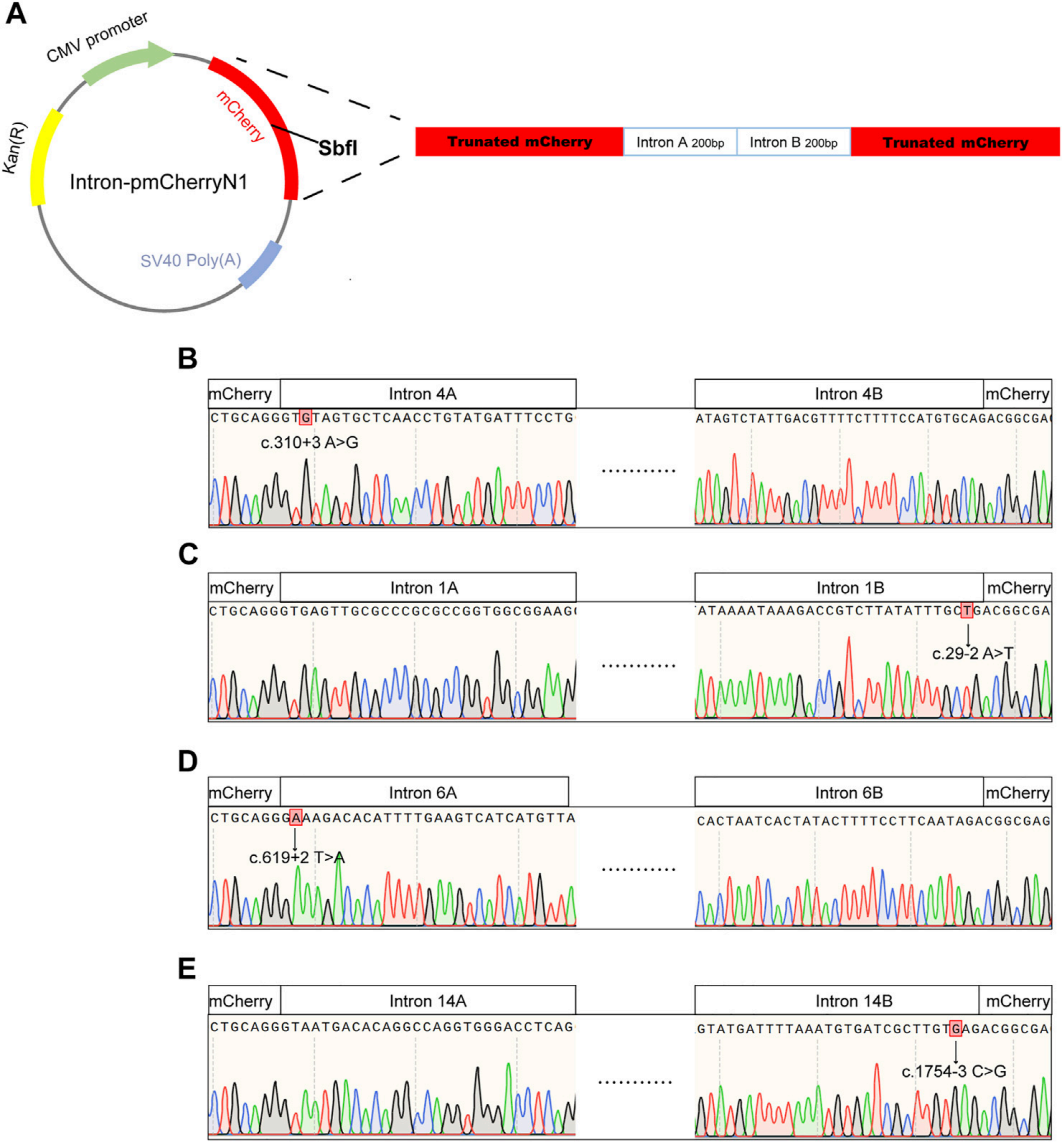
Sequences used for *in vitro* RNA splicing assays. **(A)** Schematic view of the pmCherryN1 plasmid used in the splicing assays; a shortened *RPGR* intron was generated for use in the assay by selecting the DNA sequence around the splice acceptor site (intron A), which was combined with the DNA sequence around the splice donor site (intron B). Sanger sequencing indicated that intron 4A contained the c. c.310 + 3A>G variant **(B)**, intron 1B contained the c.29-2A>T variant **(C)**, intron 6A contained the c.619 + 2T>A variant **(D)**, and intron 14B contained the c.1754-3C>G variant **(E)**.

**FIGURE 6:**
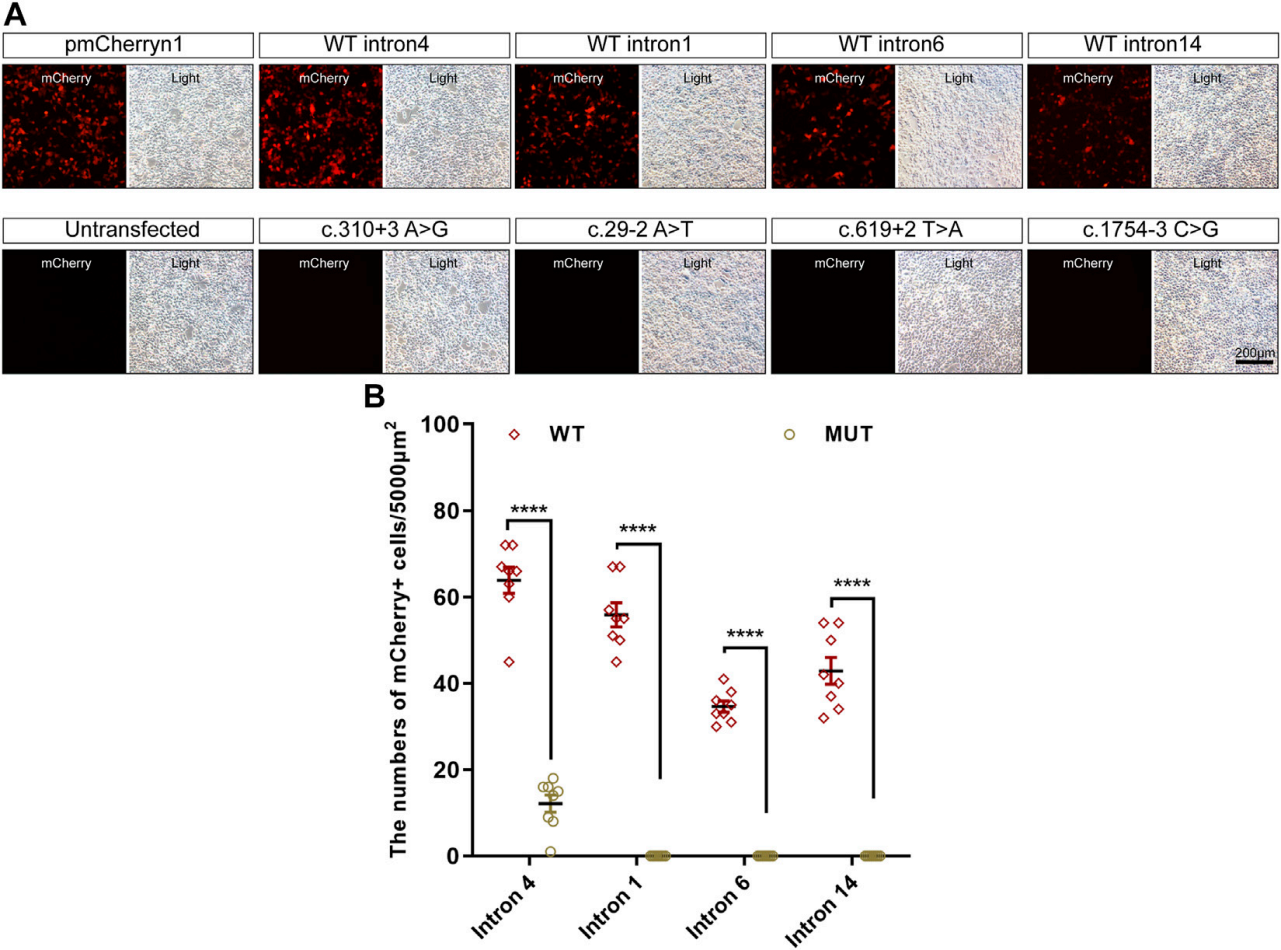
Expression of mCherry in *in vitro* splicing assays. **(A)** mCherry expression in HEK293T cells transfected with the pmCherryN1 vector containing WT or mutant introns, 24 h after transfection; scale bar = 200 μm. **(B)** The number of mCherry+ cells per 5,000 μm^2^ in HEK293T cells transfected with mutant-intron-pmCherryN1 plasmids was statistically lower than that in 293T cells with WT-intron-pmCherryN1 plasmids. Error bars indicate the SEM, and the significance was calculated by a two-tailed unpaired t-test. ****p < 0.001.

Intronic variants, including c.310 + 3A>G, c.29-2A>T, c.619 + 2T>A, and c.1754-3C>G, have been previously reported to be disease-causing (Buraczynska et al., 1997; Wang et al., 2018; Liu et al., 2021). Splice prediction analysis using HSF indicated that the pathogenic variants c.310 + 3A>G and c.619 + 2T>A may influence the donor splice site, whereas c.29-2A>T and c.1754-3C>G may influence the acceptor splice site (Supplementary Data). Additionally, these intronic pathogenic variants were investigated using *in vitro* splicing assays. Shortened versions of introns 4, 1, 6, and 14 were created for the splice assay (Figure 5). A wild-type (WT) or mutant variant of the shortened intron was created and inserted into the mCherry coding sequence of a reporter construct (Figure 5A). Sanger sequencing results of reporter plasmids with the four intronic variants are shown in Figures 5B–E. If not successfully spliced, the presence of the intron would result in a premature stop codon and therefore would not lead to mCherry expression. Successful splicing would enable the complete removal of the intron and would therefore lead to mCherry expression. As illustrated in Figure 6A, mCherry expression was observed in HEK293T cells transfected with WT-intron-pmCherryN1, whereas mCherry expression was not observed in HEK293T cells transfected with mutant-intron-pmCherryN1. After 72 h of transfection, the number of mCherry+ cells/5,000 μm^2^ in HEK293T cells transfected with mutant-intron-pmCherryN1 plasmids was statistically (*p* < 0.001) lower than that in 293T cells transfected with WT-intron-pmCherryN1 plasmids (Figure 6B), indicating that these four intronic variants disrupted *RPGR* splicing.

## Discussion

This study describes the natural history of 34 RP cases and 7 CORD cases associated with *RPGR* mutations in a large Chinese cohort (40 male probands and 1 female proband from 41 different families), thereby confirming that the inheritance mode of these families was XL. We found that 70.73% (29/41) of the cases carried *RPGR* ORF15 mutations. All *RPGR* mutations causing a CORD phenotype were found in exon ORF15. In this study, we identified 34 disease-causing mutations, of which one was novel and 21 were first reported in our previous study (Liu et al., 2021). These results expand the IRD mutation spectrum and provide new targets for the diagnosis and treatment of IRD.

Previously, when patients were diagnosed with XL IRDs, PCR-based Sanger sequencing was used directly to screen the disease-causing mutations in *RPGR* and *RP2* for these patients (Yang et al., 2014), because the majority of XLRP cases are attributed to mutations in *RPGR* (30%–80% of XLRP patients (Vervoort et al., 2000; Pelletier et al., 2007)) and *RP2* (7%–18% of XLRP patients (Sahel et al., 2014)). However, as a result of family planning over the past 30 years in China, some patients are the only child in a family, making it impossible to determine the exact inheritance pattern and recurrence risk in offspring. In this cohort, we found that 18 families had sporadic inheritance. A previous study (Martin-Merida et al., 2019) of a large cohort of 877 cases of sporadic retinitis pigmentosa (sRP) showed that 84.5% (279/330) of the solved sRP cases carried homozygous or compound heterozygous variants in the AR gene. This result indicates that the majority of sRP cases have an AR inheritance. In this cohort, genetic testing showed that 18 sporadic cases were revised as XL inheritance, which may result from the following facts: 1) the parents of these probands were unaffected and did not carry any disease-causing variant whereas the probands were affected by a new pathogenic mutation; and 2) the mother and the other female elders, but no males, carried a disease-causing variant, resulting in the affected probands inheriting this variant from their mother.

Despite the XL inheritance of *RPGR*, typical RP characteristics were observed in female carriers (Wu et al., 2010; Nanda et al., 2018; Salvetti et al., 2021). Therefore, the XL RP cases might be mistaken as having an AD inheritance mode, resulting in an incorrect prediction of recurrence risks and errors in patient prognosis. Previous studies reported that mutations in exons 1–14 and ORF15 can result in severe RP phenotypes in female carriers (Nanda et al., 2018). In this cohort, several female carriers in two families with a clinical diagnosis of AD RP carried c.3001G>T (p. Glu945*) and c.2730_2731del (p. Glu911Glyfs*167) in the *RPGR* ORF15. The p. Glu945* mutation was first reported in our previous study of family P19. Different from her two affected sisters, individual III:3, who had this heterozygous mutation, was unaffected, indicating that, in a given family, the same *RPGR* mutation can result in different phenotypes, even in female carriers. In family P27, c.607G>C (p. Ala203Pro) caused typical RP phenotypes in the proband, but not in individual III:3. Previous studies (Aguirre et al., 2002; Koenekoop et al., 2003; Nanda et al., 2018; Kurata et al., 2019; Salvetti et al., 2021) showed that the intrafamilial spectrum of severity in female carriers of XL retinal disorders partially resulted from X-chromosome inactivation. Based on the *Drosophila* model, Dobyns (2006) proposed a penetrance and severity index for X-linked diseases, as opposed to the terminology of X-linked dominant, semidominant, or recessive inheritance. Random X-inactivation can result in a mosaic pattern of cells expressing WT and mutant genes (Lyon, 2002). Although a previous study (Talib et al., 2018) indicated that random X-inactivation cannot fully explain the clustering of nearly complete penetrance in several families based on their investigation of the phenotypes of 125 female carriers of phenotypic variants in *RPGR*, the intrafamilial variability of severity is likely to be due to genetic and/or environmental modifiers as well as potentially skewed X inactivation. Previously, a large number of *RPGR* variants were reported to result in a Severe “Male Pattern” in female carriers (Nanda et al., 2018; Salvetti et al., 2021) and variants c.3001G>T (p. Glu945*), c.2730_2731del (p. Glu911Glyfs*167), and c.607G>C (p. Ala203Pro) were reported to be associated with RP phenotypes in female carriers. A previous study (Talib et al., 2018) also indicated that 23% of female *RPGR* carriers displayed complete RP or COD/CORD phenotypes, and our study also found several female carriers with RP, indicating that affected female *RPGR* carriers are not uncommon. Even in a single family, patients with the same variants may have different phenotypes, for example, in family P19, individual III:4, a female carrier, also suffered from RP, whereas III:1 was unaffected, illustrating the clinical heterozygosity of IRD and emphasizing the significance of genetic testing. Currently, several gene therapy trials for *RPGR*-XLRP male patients are ongoing or have been completed (https://clinicaltrials.gov), and affected female carriers should be recruited for, and may benefit from, gene therapy.

*RPGR* ORF15 is a hotspot for pathogenic mutations (Vervoort et al., 2000; Breuer et al., 2002) and splice site pathogenic variants are uncommon within any part of the *RPGR* (Cehajic-Kapetanovic et al., 2020). The pathogenicity of c.310 + 3A>G, c.29-2A>T c.619 + 2T>A, and c.1754-3C>G has been validated in previous studies (Buraczynska et al., 1997; Wang et al., 2018; Liu et al., 2021). However, few functional studies have validated the pathogenicity of the intronic variants of *RPGR*. The splicing process is a complex event that is important for proper protein synthesis, and splicing mutations may occur in introns and exons, which disrupt existing splice sites, create new sites, or activate cryptic sites (Anna and Monika, 2018). The most common mutations affect +1 and +2 residues at the 5′ donor splice site and −1 and −2 residues at the 3′ acceptor splice site (Anna and Monika, 2018). There are a large number of online tools that can be used to predict the pathogenicity of splicing variants (Anna and Monika, 2018); however, the exact splicing effect should be tested at different levels: DNA, RNA, RNA-protein interactions, or at the protein level (Thery et al., 2011; Fredericks et al., 2015). Analysis of RNA extracted from a patient’s cells or tissues is the simplest and most effective method to determine whether the selected variant affects splicing (Anna and Monika, 2018). In this study, RNA from the patients’ retinal tissues was not available. Therefore, the minigene assay may be an alternative method. In the minigene assay, an amplified fragment of an analyzed gene, such as a specific exon with surrounding intronic sequences with and without mutations, is cloned into a special expression plasmid, enabling the analysis of pre-mRNA splicing. Because of the large size of *RPGR* introns, we developed a simplified molecular assay in which we designed reporter constructs to investigate the effect of variants on RNA splicing *in vitro*, according to a previous study (Cehajic-Kapetanovic et al., 2020). By comparing the mutant and WT constructs, we found that a single nucleotide variation influenced splicing, also indicating the practicability of our approach, which can be used to validate splicing effects and validate which exons would be skipped.

In summary, the findings in this cohort provide useful insights for the genetic and clinical counseling of XL RP and CORD patients, which would be useful for ongoing and future gene therapy trials. Herein, we also describe the genotype and phenotype correlations of several female *RPGR* carriers who showed a male phenotype, reminding us of the significance of genetic testing in this scenario. Our study highlights the potential value of a molecular splice assay to confirm a new disease-causing intronic variant of *RPGR*.

## Data Availability

The original contributions presented in the study are included in the article/Supplementary Material. further inquiries can be directed to the corresponding authors.
