# Supplementary material for: Analysis of RPGR gene mutations in 41 Chinese families affected by X-linked inherited retinal dystrophy
Source: Front Genet. 2022 Oct 6;13:999695. doi: 10.3389/fgene.2022.999695 (PMC9582779; doi:10.3389/fgene.2022.999695)
Supplement: Supplementary file 1 [file DataSheet1.PDF]

Supplemental Data

Analysis on *RPGR* gene mutations in 41 Chinese families affected with X-linked inherited retinal dystrophies

Xiaozhen Liu, Ruixuan Jia, Xiang Meng, Likun Wang and Liping Yang

Gene: ***RPGR***
Chromosome position: X:38321024-38321024 (GRCh38)
Mutation: **X 38321024 T/C**
Nomenclature per transcript:

Transcript (Ensembl)	Nomenclature (HGVS)
ENST00000339363.7	c.310+3A>G
ENST00000642395.2	c.310+3A>G
ENST00000642558.1	c.217+3A>G
ENST00000644238.1	c.310+3A>G
ENST00000644337.1	c.310+3A>G
ENST00000645032.1	c.310+3A>G
ENST00000647261.1	c.310+3A>G

Pathogenicity prediction on splicing signals (HSF Pro system from Genomnis, https://genomnis.com)

The mutation disrupts the wild type donor splice site, it most probably affects splicing

Algorithm: **HSF Donor site** (matrix GT)
Donor site position: chrX:38321029
Wild type sequence: CAGGTATAG
Mutant sequence: CAGGTGTAG
Splice site strength: 80.64>62.19 (**-22.88%**)

The modification between the wild type and the mutant sequence is above the threshold of 10%, thus indicating a strong impact on the splice site.

Algorithm: **MaxEnt Donor site**
Donor site position: chrX:38321029
Wild type sequence: CAGGTATAG
Mutant sequence: CAGGTGTAG
Splice site strength: 8.73>3.1 (**-64.49%**)

The modification between the wild type and the mutant sequence is above the threshold of 30%, thus indicating a strong impact on the splice site.

CONCLUSION
The mutation is predicted to disrupt the donor splice site of the exon #4 of the *RPGR* gene. For such mutation the impact is usually the skipping of the exon (note that for some mutations a cryptic splice site may also be used).
The exon 4 is an in-frame exon so its skipping may produce an in-frame transcript that may still code for a protein.
Nevertheless, as this codon encodes a part of the RCC1 protein domain, **the c.310+3A>G mutation may be annotated as a pathogenic mutation**.

Gene: ***RPGR***
Chromosome position: X:38323526-38323526 (GRCh38)
Mutation: **X 38323526 T/A**
Nomenclature per transcript:

Transcript (Ensembl)	Nomenclature (HGVS)
ENST00000642395.2	c.29-2A>T
ENST00000642558.1	c.29-2A>T
ENST00000644337.1	c.29-2A>T
ENST00000645032.1	c.29-2A>T
ENST00000647261.1	c.29-2A>T

Pathogenicity prediction on splicing signals (HSF Pro system from Genomnis, https://genomnis.com)

The mutation disrupts the wild type acceptor splice site, it most probably affects splicing

Algorithm: **HSF Donor site** (matrix GT)
Acceptor site position: chrX: 38323536
Wild type sequence: TTATATTTGC**A**GAT
Mutant sequence: TTATATTTGC**T**GAT
Splice site strength: 90.42>62.55 (**-30.82%**)

The modification between the wild type and the mutant sequence is above the threshold of 10%, thus indicating a strong impact on the splice site.

Algorithm: **MaxEnt Donor site**
Donor site position: chrX: 38323544
Wild type sequence: AGACCGTCTTATATTTGC**A**GATT
Mutant sequence: AGACCGTCTTATATTTGC**T**GATT
Splice site strength: 7.96>-0.4 (**-105.03%**)

The modification between the wild type and the mutant sequence is above the threshold of 30%, thus indicating a strong impact on the splice site.

CONCLUSION
The mutation is predicted to disrupt the acceptor splice site of the exon #2 of the *RPGR* gene. For such mutation the impact is usually the skipping of the exon (note that for some mutations a cryptic splice site may also be used).
The exon 2 is an in-frame exon so its skipping may produce an in-frame transcript that may still code for a protein.
The amino acids encoded by this exon do not mat into a protein domain, so the consequence of this mutation is more difficult to predict at the protein level. Therefore **the c.29-2A>T mutation may be annotated as a probably pathogenic mutation**.

Gene: ***RPGR***
Chromosome position: X:38317314-38317314 (GRCh38)
Mutation: **X 38317314 A/T**
Nomenclature per transcript:

Transcript (Ensembl)	Nomenclature (HGVS)
ENST00000642395.2	c.619+2T>A
ENST00000642558.1	c.526+2T>A
ENST00000644337.1	c.619+2T>A
ENST00000645032.1	c.619+2T>A
ENST00000647261.1	c.619+2T>A

Pathogenicity prediction on splicing signals (HSF Pro system from Genomnis, https://genomnis.com)

The mutation disrupts the wild type donor splice site, it most probably affects splicing

Algorithm: **HSF Donor site** (matrix GT)
Donor site position: chrX: 38317318
Wild type sequence: CAAG**T**AAGA
Mutant sequence: CAAG**A**AAGA
Splice site strength: 87.24>60.1 (**-31.11%**)

The modification between the wild type and the mutant sequence is above the threshold of 10%, thus indicating a strong impact on the splice site.

Algorithm: **MaxEnt Donor site**
Donor site position: chrX: 38317318
Wild type sequence: CAAG**T**AAGA
Mutant sequence: CAAG**A**AAGA
Splice site strength: 7.61>-0.58 (**-107.62%**)

The modification between the wild type and the mutant sequence is above the threshold of 30%, thus indicating a strong impact on the splice site.

CONCLUSION
The mutation is predicted to disrupt the acceptor splice site of the exon #6 of the *RPGR* gene. For such mutation the impact is usually the skipping of the exon (note that for some mutations a cryptic splice site may also be used).
The exon 6 is an in-frame exon so its skipping may produce an in-frame transcript that may still code for a protein.
The amino acids encoded by this exon do not mat into a protein domain, so the consequence of this mutation is more difficult to predict at the protein level. Therefore **the c.619+2T>A mutation may be annotated as a probably pathogenic mutation**.

Gene: **RPGR**
Chromosome position: X:38287248-38287248 (GRCh38)
Mutation: **X 38287248 G/C**
Nomenclature per transcript:

Transcript (Ensembl)	Nomenclature (HGVS)
ENST00000642395.2	c.1754-3C>G
ENST00000644337.1	c.1568-3C>G
ENST00000645032.1	c.1754-3C>G

Pathogenicity prediction on splicing signals (HSF Pro system from Genomnis, https://genomnis.com)

The mutation disrupts the wild type acceptor splice site, it most probably affects splicing

Algorithm: **MaxEnt Acceptor site**
Acceptor site position: chrX: 38287265
Wild type sequence: TAAATGTGATCGCTTGT**C**AGAGA
Mutant sequence: TAAATGTGATCGCTTGT**G**AGAGA
Splice site strength: 4.19>-9.06 (**-316.23%**)

The modification between the wild type and the mutant sequence is above the threshold of 30%, thus indicating a strong impact on the splice site.

CONCLUSION
The mutation is predicted to disrupt the acceptor splice site of the exon #14 (ENST00000644337.1) or #15 (ENST00000642395.2 and ENST00000645032.1) of the *RPGR* gene. For such mutation the impact is usually the skipping of the exon (note that for some mutations a cryptic splice site may also be used).
The exon 14/15 is an out-of-frame exon so its skipping will most probably result in the absence of the RPGR protein (null allele) through the activation of the NMD (Nonsense Mediated Decay). Therefore **the c.1568-3C>G mutation may be annotated as a pathogenic mutation**.

Table 1 Primers used in this study.

Primers	Forward (5'-3')	Reverse (5'-3')
RPGR exon1	AACCGTCCTCTACAGCCTC	CTCTTTCCCGTTCTCCCC
RPGR exon2-3	GGAAGGCTTAAACATTGCCA	TGGGGGATATTCAAATGCAA
RPGR exon4	CTGAAAACCTCTGGTTTGCT	TGCAAAGGCAAACGTGTACT
RPGR exon5	CCTTGCTTGTTTTGCTTTAT	TTCGGTTTACTGAGTTGGC
RPGR exon6	CAATCAGGCTGTTCTGTGTTC	CTGAGAAAGTCCCACCAAAG
RPGR exon7	ATCTCAAGAAAGGTCAAATGTA	TAATAAAATGGTGGTCGCC
RPGR exon8	TTTTCCCCAGAGGCACTTA	TTCTGACATCATCGGCCTAT
RPGR exon9	TTACATGCAGGACCACAGAGA	AAAGGAAGAGGCTAAAGGAGG
RPGR exon10	GATTCACCAAGCCAGTCTGT	AGCACCATTGTCTATATGCAA
RPGR exon11	AATGTTGTGGAGTGTTGGCA	AGGATATTCCCGGATTTGAG
RPGR exon12-13	TCAATTTCCCTGACATGAGG	CCAAAGAGCAAATTTCAGCA
RPGR exon14	GAGAGTGGCACAAATGATCCT	TGTCCTCCATCACTTTCCTTT
RPGR exon15	TGATGAAGTGGAAACTGACC	CCTCATCTTGCCAGTGTTCT
RPGR exon16	CAGCAATATCAAATCCCTCG	TTCACAAATAAGCCAAAGCC
RPGR exon17	GGACATTTGCAATCAGTCAG	ATAGCTTGTTATGAGGATCATG
RPGR exon18	GGGTGAGCTCTTTTCGTATTA	CAAATGCTAACCAAAGGAAAG
RPGR exon19	ATCCTGACTGCCTTTTGGT	GCCACAACACTTTAGGGAGA
